# Penetration Depth of a Soil Moisture Profile Probe Working in Time-Domain Transmission Mode †

**DOI:** 10.3390/s19245485

**Published:** 2019-12-12

**Authors:** Marcin Kafarski, Jacek Majcher, Andrzej Wilczek, Agnieszka Szyplowska, Arkadiusz Lewandowski, Alicja Zackiewicz, Wojciech Skierucha

**Affiliations:** 1 Institute of Agrophysics, Polish Academy of Sciences, Doświadczalna 4, 20-290 Lublin, Poland; a.wilczek@ipan.lublin.pl (A.W.); a.szyplowska@ipan.lublin.pl (A.S.); w.skierucha@ipan.lublin.pl (W.S.); 2 Department of Electrical Engineering and Electrotechnologies, Lublin University of Technology, Nadbystrzycka 38A, 20-618 Lublin, Poland; j.majcher@pollub.pl; 3 Institute of Electronic Systems, Warsaw University of Technology, Nowowiejska 15/19, 00-665 Warsaw, Poland; a.lewandowski@elka.pw.edu.pl; 4 Institut für Ökologie, Technische Universität Berlin, Ernst-Reuter-Platz 1, 10587 Berlin, Germany; a.zackiewicz@tu-berlin.de

**Keywords:** soil moisture, profile probe, TDT probe, electromagnetic simulations

## Abstract

Soil moisture is one of the most important soil parameters. Knowledge of volumetric water content (VWC) of the root zone as well as the VWC dynamics in the soil profile is especially important for agriculture. Monitoring VWC at several depths in the soil profile can be performed using several soil moisture sensors placed at various depths. However, the use of a profile probe is more convenient, because the installation of a single probe is less disturbing to the soil, as well as less laborious and more cost-effective. The objective of the paper is to present the design and performance of a novel profile probe working in the time-domain transmission mode (P-TDT probe) with emphasis put on the penetration depth and sensitivity zone. The performance of the probe was assessed with the use of finite element method (FEM) simulations in the frequency domain, transient simulations in the time domain and laboratory experiments with the use of a vector network analyzer (VNA) working in the 10 MHz–10 GHz frequency range. It was concluded that the effective soil volume measured by the profile probe of a given geometry is equivalent to a soil thickness of about 20 mm around the tested probe. The internal part of the probe body had a negligible effect on the measurement results, as it does not change with soil moisture. Moreover, the transmitted signal amplitude was related to the soil electrical conductivity.

## 1. Introduction

Commonly used practice for the measurement of soil volumetric water content (VWC) relies on point measurement data for the analysis of water flow dynamics caused by evaporation, transpiration and precipitation, as well as suction from groundwater resources [1]. Installation of individual sensors at selected depths is destructive to the soil, the cost of the multiple-probe setup together with the necessary accessories (cables, interfaces, data-loggers, etc.) may be substantial, and the installation in the field of a system consisting of many elements is laborious and prone to generate measurement errors caused by possible carelessness during probe installation. Therefore, if the knowledge of VWC at several depths in a soil profile is needed, it is more effective and convenient to use a profile probe in the form of a single tube with sensing elements placed at various distances along the tube [2]. Such probes usually measure VWC together with soil bulk electrical conductivity (*EC_b_*) and temperature (*T*) at different depths of a soil profile. Soil disturbance is minimal and usually the total cost of measurement in three to four soil layers is substantially smaller than is the case with the installation of several separate probes.

Neutron moisture meters (NMM) had been successfully used for the purpose of determining the VWC in the soil profile for nearly 50 years, but radiological regulations and the necessity for constant supervision of the NMM forced soil scientists to use other measurement equipment [3,4,5]. The use of soil electromagnetic (EM) properties gave rise to new sensors and meters of soil VWC. They perfectly fulfill the requirement for automatic and safe measurements. The measurement principle of EM sensors is based on the fact that the presence of water modifies soil complex dielectric permittivity, which in turn modifies the propagation time of EM waves (which is the property used in time-domain sensors), as well as the capacitance/impedance (and oscillation frequency) of a capacitor with soil as the dielectric (which is the property used in frequency-domain sensors). The cost of EM measurement equipment for VWC differs significantly with the measurement frequency range and the measurement accuracy. The GHz frequency sensors do not require soil specific calibration for the majority of mineral soils, while low-frequency sensors (<100 MHz, especially) are susceptible to measurement errors due to electrical conductivity and interfacial polarization effects [4,5,6]. The increasing demand for EM sensors of soil VWC in the global market has resulted in a growing supply in products of various quality, differing in measurement accuracy, calibration methods and measurement volume etc., giving rise to confusion among the users, this demonstrates the need for international testing standards of EM sensors for VWC [7]. The most important factor in the performance of VWC profile probes is the measurement volume or the sphere of influence. The soil volume measured by the neutron probe is much larger than in the case of any EM probe. Roughly, it is a sphere with a radius from 15 cm for saturated soil to 50 cm for dry soil. Measurements close to the soil surface also have a greater probability of error due to the influence of the soil/air interface [8]. A smaller measurement volume with EM sensors minimizes the effect of this source of error, however, the effect of the air gap between the access tube and soil caused by possible carelessness during installation or shrinking of the soil can significantly affect the measured VWC values. Moreover, most profile probes have a very small measurement volume, smaller even than individual probes installed at separate depths [4].

The radius of the sphere of influence depends in general on the sensor design. The maximum possible radius of this sphere can be estimated based on the penetration depth $d_{pmax}\left(m\right)$ of the plane wave, which is given as [9]:

(1) $$d_{pmax}=\frac{c}{2\pi f\sqrt{2\varepsilon^{\prime }\left[\sqrt{1+{\left(\frac{\varepsilon^{\prime \prime }}{\varepsilon^{\prime }}\right)}^{2}}-1\right]}},$$

where $\varepsilon^{\prime }$ and $\varepsilon^{\prime \prime }$ are the values of the real and imaginary parts of the complex relative dielectric permittivity $\varepsilon^{*}=\varepsilon^{\prime }-j\varepsilon^{\prime \prime }$ of the tested material, respectively, *c* is the speed of light in free space and *f* is the frequency of the electromagnetic wave. The imaginary part of dielectric permittivity depends on dielectric loss $\varepsilon_{d}$ and bulk electrical conductivity $EC_{b}$ (dSm^−1^), according to the relation $\varepsilon^{\prime \prime }=\varepsilon_{d}+\frac{EC_{b}}{2\pi f\varepsilon_{0}}$. Therefore, the $d_{pmax}$ value decreases with an increase in frequency and an increase in bulk electrical conductivity of the measured material. The values of the penetration depth of the plane wave may reach an order of meters at frequencies below 100 MHz, and decrease to centimeters above 10 GHz.

**Figure 1:**
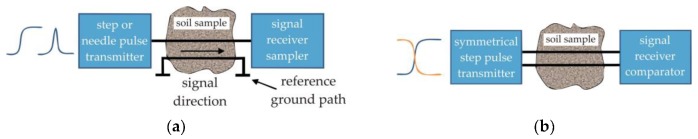
Innovation of the soil moisture profile probe based on the time-domain transmissometry technique (P-TDT) is based on using a patented method of symmetrical signal paths: (**a**) traditional fast signal processing requires expensive pulse transmitters and receiver samplers and (**b**) application of fast digital circuits with symmetrical signals are less sensitive to impedance mismatches in the signal path.

In the paper, an innovative soil moisture profile probe based on the time-domain transmissometry technique (P-TDT probe) is presented. The crucial innovation of the P-TDT probe is the application of digital fast integrated circuits and two symmetrical, opposite phase, electrical step pulses for the generation and measurement of their travel time t along a transmission line placed in soil (see Figure 1a,b). Analog signal processing of fast step or needle electrical pulses require individually calibrated samplers. The use of symmetrical transmission allows for the improvement of the transmission time measurement accuracy as it is less sensitive to impedance mismatches of the signal paths. The use of symmetrical transmission also reduces the total cost of a single segment of the proposed P-TDT probe due to the application of highly integrated ECL digital circuits. The technical details of this solution are described in the Polish patent No. PAT.228416 [10].

The objective of the paper is to determine the penetration depth $d_{p}$ of the presented P-TDT probe with the use of digital simulations and broadband measurements (10 MHz–10 GHz) using a vector network analyzer (VNA) and a profile probe prototype. A minor part of the research work presented here was published in [11].

## 2. Materials and Methods

The digital simulations were performed with the use of the following software tools: (1) 3D electromagnetic wave simulation in the frequency domain for finding the electromagnetic interaction between the probe body and the surrounding material, and (2) electronic design software to convert data collected in the frequency domain to the time domain. This combination of simulations helped to significantly shorten the time to develop a working prototype of the profile probe by optimizing its geometry and measurement circuitry, to identify sources of errors, to test the resolution/accuracy and to minimize the cost of the measurement unit.

Below, the mechanical construction of the prototype P-TDT probe is described. Its geometry was analyzed and optimized using 3D finite element method (FEM) simulations. The geometry chosen for the P-TDT prototype was tested with respect to the penetration depth of the electromagnetic pulse in the dielectric media simulating soil of various moisture content and salinity. The penetration depth of the prototype was verified based on measurements in ethanol, distilled water and sandy soil with two moisture values. All calculations were made using Excel spreadsheet functionality, including nonlinear curve fitting by Solver add-in.

### 2.1. Construction of the P-TDT Probe

**Figure 2:**
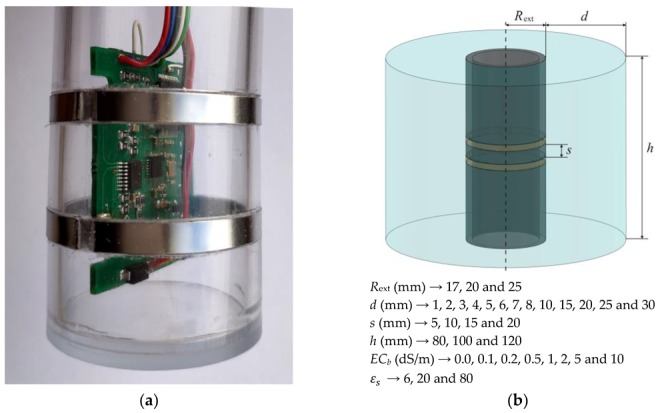
The shape and dimensions of one section of the TDT profile probe prototype: (**a**) general idea of the TDT profile probe construction with the initial version of electronics and (**b**) dimensions of one section of the TDR probe and variation of the materials’ dielectric parameters: *EC_b_* and $\varepsilon_{s}$ used in digital simulations (see Equation (2)).

A prototype of a single P-TDT probe section is shown schematically in Figure 2. It was constructed as a transmission line, which was wound around the plastic body of the P-TDT supporting tube.

The probe was designed to be placed vertically in the soil in a previously drilled pilot hole without an access tube. The entire profile-probe consists of a number of transmission-line sections such as the one shown in Figure 2a. The number of sections may vary so as to monitor soil moisture, temperature and electrical conductivity at required soil depths.

The electromagnetic field generated by the pulse travelling along a section with the ‘parallel’ transmission line penetrates the soil and the internal part of the PVC tube. The internal part of the probe is watertight and consists of: material forming the body of the probe (preferably not absorbing water from the surroundings, e.g., POM-C [12]), air, a printed circuit board (PCB) made of epoxy resin type FR4 and electronics. The internal part of the probe presents a stable environment for electromagnetic wave penetration. On the opposite side, the soil placed outside the probe body changes the conditions for fast electrical pulse travel time and attenuation due to the variation of its moisture content, temperature and electrical conductivity.

The physical quantity used to describe electrical properties of the soil from the point of view of the electromagnetic field generated by the propagating electric pulse, is its relative complex dielectric permittivity $\varepsilon^{*}$. The sensing element consists of two metal strips wound around the probe cylindrical enclosure (Figure 2a). In the presented design, the sensing element is in direct contact with the soil. Three or more identical sections in the probe are built from parallel transmission lines that are equipped with independent electronic circuits. Each section monitors soil moisture, temperature and electrical conductivity at required soil depths. Figure 2b shows the shape and dimensions of one section of the digitally modeled probe and its surrounding material.

The electromagnetic field generated by the pulse travelling along the transmission line penetrates the soil and the internal part of the plastic tube. The internal part of the probe consists mainly of the plastic body of the probe, air as well as an electric board (PCB made of epoxy resin type FR4) with electronics that as a whole present a stable environment for electromagnetic wave penetration. Only the soil at the external part of the transmission line causes the electromagnetic field to change with moisture content, temperature and electrical conductivity. Relative complex dielectric permittivity $\varepsilon^{*}$ is related to electric pulse travel time *t* along the transmission line, $t=f\left(\varepsilon^{*}\right)$.

### 2.2. Digital Simulations

Digital simulations combine the frequency and time domain analysis of a broadband signal travelling along the metallic strips forming the transmission line of the profile probe. Frequency domain analysis was done by means of a three dimensional electromagnetic simulation software pack (Keysight EMPro 3D EM Simulation Software). It determined electromagnetic interaction between the probe’s surrounding material and the body of the probe in the frequency range from 10 MHz to 10 GHz. This interaction is described by the ***S*** scattering matrix, which was calculated using the finite element method (FEM) [13]. The Port1 (input) and Port2 (output) were placed at the beginning and the end of the two metallic strips respectively. The boundary conditions of the material were fixed as absorptive, thus no reflection from boundaries occurred. The material outside the measured thickness had dielectric parameters of air ($\varepsilon^{*}=1-j0$).

Next, the scattering matrix ***S*** was imported into the Keysight Advanced Design System software pack (ADS) as an element of the electric circuit so as to convert the frequency domain simulated data into the time domain. The ADS software calculates the propagation time and the attenuation of an electric pulse (narrow symmetrical needle pulse of 1 V amplitude and the half-width of 150 ps) along the waveguide formed from the metallic strips wound around the supporting plastic tube of the profile probe. These values depend on the dielectric permittivity, which is related to the volumetric water content (VWC), bulk electrical conductivity ($EC_{b}$) and volume of the soil material surrounding the profile probe.

The dielectric parameters of the tested material were described by a single-relaxation Debye formula:

(2) $$\varepsilon^{*}=\varepsilon_{\infty }+\frac{\varepsilon_{s}-\varepsilon_{\infty }}{1+j2\pi f\tau }+j\frac{EC_{b}}{2\pi f\varepsilon_{0}},$$

where $\varepsilon_{\infty }=$ 5.2 and τ= 9.45 × 10^−12^ s are values for water, $\varepsilon_{0}$ = 8.85 × 10^−12^ Fm^−1^, applied frequency *f* changed from 10 MHz to 10 GHz with at least 20 points per decade. The parameters that varied were: *R*_ext_ (mm)—external radius of the plastic tube with metal strips of the probe’s transmission line, *d* (mm)—thickness of the probe’s surrounding material, $EC_{b}$ (dSm^−1^) and $\varepsilon_{s}$—its electrical conductivity and low frequency dielectric permittivity respectively, *s* (mm)—distance between two metal strips of the probe’s parallel transmission lines and *h* (mm)—height of the profile probe section. The width and thickness of metal strips were fixed to 5 mm and 0.3 mm respectively.

**Figure 3:**
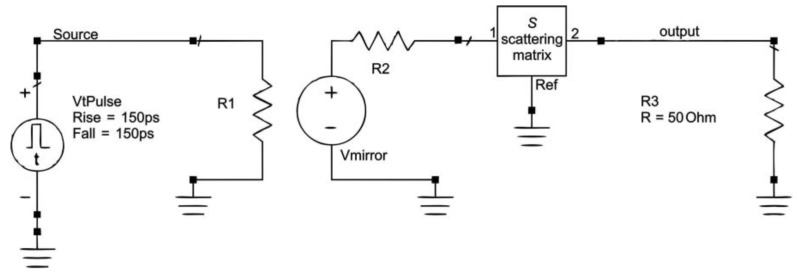
Elementary electrical circuit used in Advanced Design System (ADS) design software [11]. The ‘output’ signal contains information about the shape and delay of the initial signal from the VtPulse generator after covering the distance along the transmission line of the profile probe represented by the scattering matrix ***S***.

The elementary electric circuit used in ADS is presented in Figure 3. The VtPulse element is a needle pulse generator of 150 ps rise and fall times and 1 V amplitude, the R1 = R2 = R3 = 50 ohm, Vmirror serves as the controlled voltage source, ***S*** is the scattering matrix imported from EMPro for the given geometry and external material of the profile probe. In total, several thousand FEM digital simulations were done for many combinations of *R*_ext_, *d*, *s*, *h*, *EC_b_* and $\varepsilon_{s}$ (see Figure 2b). The use of the R1, R2 and Vmirror setup in the circuit enabled connection to the source VtPulse in parallel so as to quickly obtain several waveforms representing various combinations of the *d* parameter when the others were fixed.

### 2.3. VNA Measurements

**Figure 4:**
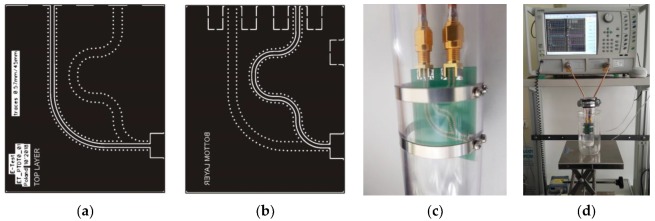
Elements of the profile probe prototype: (**a**) top and (**b**) bottom layer of the printed circuit board (PCB) made of four-layer FR4 epoxy laminate as the 50 ohm interface between vector network analyzer (VNA) ports and metal strips of the probe; (**c**) single section of the prototype profile probe for VNA measurements of the scattering matrix ***S*** and (**d**) complete measurement setup for the experimental verification of the effective penetration depth of the P-TDT profile probe.

Verification of the profile probe performance obtained from digital simulations was done on the prototype construction presented in Figure 4. The two internal layers of the FR4 four layer printed circuit board (PCB) were connected to the ground by means of via pads. The thickness of the top and bottom layer was the same, and together with the top and bottom paths they formed 50-ohm transmission lines of the same length (Figure 4a,b). The lower metallic strip of the profile probe was connected to VNA port 1 by means of the transmission line on the bottom side of the PCB, and the other end of the lower strip was connected to VNA port 2 using the transmission line on the top side of the PCB. Both transmission lines were connected to the VNA ports by SMA edge female connectors and semi-rigid cables about 20 cm long (Figure 4c,d). The upper metallic strip of the profile probe was connected to the ground layer on both sides. The bottom part of the plastic tube supporting the metallic strips was tightly sealed. In this way the VNA transmission measurements were possible in materials surrounding the strips from outside. Metallic strips were made of stainless steel (width 5 mm, thickness 0.3 mm), the distance between them was *s* = 16 mm, the external and internal radius of the plastic tube were 20 mm and 17 mm respectively. The strips were connected to the PCB with screws.

The VNA (2-port Anritsu VectorStar MS4642A, bandwidth 70 kHz–20 GHz) measurements were made in a constant temperature 20 ± 0.5 °C in laboratory conditions. Before measurements the VNA was turned on for about one hour to stabilize its temperature, both ports were calibrated following the respective instructions supplied by the producer of the device. The full scattering matrix ***S*** in the frequency range 10 MHz–10 GHz was collected for each measurement. Next, the measured ***S*** parameters were imported to the Keysight ASD program for further processing in a similar way as described earlier.

The P-TDT prototype probe was placed centrally in cylindrical containers of 50 mm, 56 mm, 66 mm, 86 mm and 103 mm diameter, which were filled with deionized water and ethanol (purity 96%). The cylinders were filled with liquids to have a distance of at least 27 mm between the metallic strips and the top/bottom of the liquid in the cylinders.

**Figure 5:**
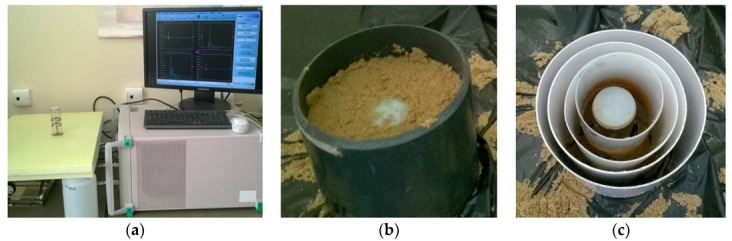
Experimental setup for soil measurements: (**a**) experimental setup; (**b**) soil measurement and (**c**) the set of pipes with different diameters.

A similar experiment with sandy soil was performed. Dielectric response of moistened soil may change depending on soil compression, thus the experimental setup was slightly modified (Figure 5) in comparison to the previous experiment with liquids in order to eliminate that error. The intention was to measure the same soil sample with the same density but with different thicknesses around the P-TDT probe.

One section of the P-TDT probe was mounted upside down in an extruded polystyrene (XPS) plate (Figure 5a) and a thin-walled pipe with 146 mm in diameter was located on the plate coaxially with the probe. The space between the pipe and the probe was filled with wet soil before performing measurements. A smaller pipe 125 mm in diameter was put into the soil coaxially with the probe (Figure 5b), then the soil outside and the bigger pipe were removed before the next measurement. The procedure was repeated six times for the pipes of 100 mm, 82 mm, 65 mm and 46 mm in diameter (Figure 5c). This method ensured measurements of the practically intact soil sample with differences between dielectric responses from each measurement resulting only from various soil-layer thickness around the P-TDT probe.

## 3. Results and Discussion

**Figure 6:**
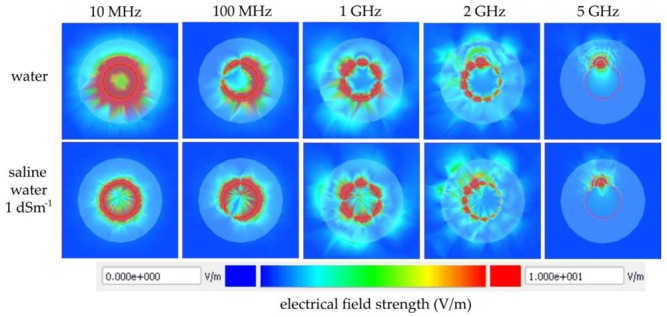
Distribution of the electromagnetic field around one section of the P-TDT probe placed in distilled water and in KCl water solution of 1 dSm^−1^ electrical conductivity in relation to the frequency of the applied signal.

Distribution of the electromagnetic field around one section of the P-TDT probe placed in distilled water and in a KCl water solution of 1 dSm^−1^ electrical conductivity in relation to the frequency of the applied signal is presented in Figure 6. These pictures are screenshots from the animation utility of the Keysight EMPro 3D electrical FEM simulations of one section of the P-TDT probe geometry from Figure 2b. Increasing the applied electrical-field frequency resulted in reduction of the penetration depth and also created resonances around the metallic strips of the probe. Above 2 GHz the strength of the electric field was continuously decreasing with frequency, and at 5 GHz the pulse could not reach the end of the P-TDT transmission line. Furthermore, the electrical conductivity of the medium limits the penetration depth of the electrical field, especially at frequencies below 100 MHz. With a frequency increase above 100 MHz there was almost no difference in the electrical field distribution in the distilled water and saline water.

**Figure 7:**
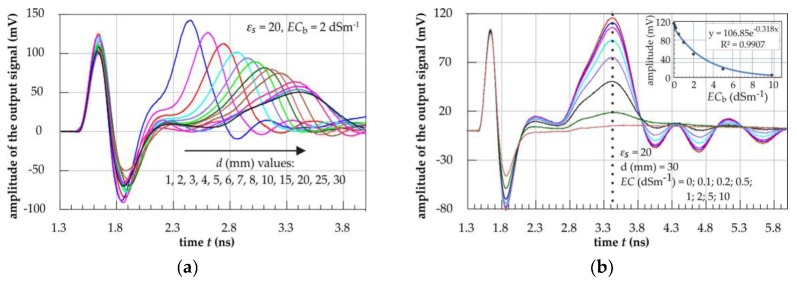
Example results in time domain as the output waveforms from Keysight ADS digital simulator (P-TDT probe geometry: *h* = 80 mm and *s* = 15 mm): (**a**) for increasing thickness of the measured material with fixed $\varepsilon_{s}$ = 20, $EC_{b}$ = 2 dSm^−1^ and (**b**) for increasing $EC_{b}$ of the measured material with fixed $\varepsilon_{s}$ = 20, *d* = 30 mm [11].

The output waveforms from the ADS digital simulator for various thicknesses *d* of the measured material with fixed values of $\varepsilon_{s}$ = 20, $EC_{b}$ = 2 dSm^−1^, *h* = 80 mm and *s* = 15 mm are presented in Figure 7a. The first positive and negative amplitude in Figure 7a,b represents a crosstalk of the initial 1 V amplitude needle pulse from the VtPulse element between input and output of the transmission line. It was caused by a capacitive coupling and may be used as the time marker to start the measurement of the propagation time of the pulse along the metallic strips of the transmission line. The increase of the material thickness *d* resulted in increasing the transmission time *t* of the applied pulse, but not in the same proportion (Figure 7a). The second positive pulse of the waveforms represents the output of the P-TDT transmission line for the material thickness equal to 1 mm, 2 mm, … and 30 mm. However, the increase of *t* was faster for smaller values of *d*. Furthermore, the amplitude of waveforms decreased as the material thickness increased. This is what was expected. Similar figures can be drawn for other combinations of $\varepsilon_{s}$, *d* and $EC_{b}$.

Increase of $EC_{b}$ for fixed values of $\varepsilon_{s}$ and *d* resulted in an exponential decrease of the transmitted signal amplitude, as seen in the inlet graph in Figure 7b. The propagation time *t* of the pulse did not change with the increase of the material $EC_{b}$, which is marked as the dotted line in Figure 7b. Above $EC_{b}$ = 5 dSm^−1^ the transmitted signal was almost completely attenuated and it was not possible to determine the transmission time *t*.

**Figure 8:**
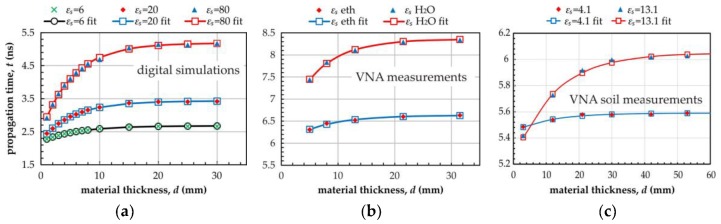
Dependence of the transmission line propagation time *t* of the P-TDT probe on the thickness *d* for three materials with various $\varepsilon_{s}$: (**a**) simulation data; (**b**) VNA measured data with the use of the P-TDT prototype presented in Figure 4c and (**c**) VNA measured data from soil measurements.

It was proposed to model the change of the propagation time *t* of the pulse along the profile probe’s transmission line with the increase of material thickness *d* with the following exponential limit formula:

(3) t=b+A(1−exp(−k·d)),

where *b* (ns) is the value of *t* for *d* = 0 mm, *A* (ns) stands for the maximum increase of *t* for *d* reaching infinity and *k* (mm^−1^) is a shape factor related to the *t* rise. The dependency of *t* on *d* given in Formula (3) was presented in Figure 8a for the digital simulation data, in Figure 8b for the VNA measurements data collected from the P-TDT prototype inserted centrally into plastic containers filled with water and ethanol and in Figure 8c for sandy soil measurements.

### 3.1. Results from Digital Simulations

Digital simulations were conducted for three materials with static values of the relative dielectric permittivity $\varepsilon_{s}$ equal to 6, 20 and 80 respectively, when material thickness *d* changed from 1 to 30 mm in steps from 1 to 5. Fitted lines follow Formula (3) with parameters given in Table 1. A new parameter *d*_99%_ was defined as the material thickness critical value for which the travel time *t* along the metallic strips of the P-TDT probe reaches the value of $t_{99\%}=0.99\;A$ and consequently $0.99\;A=A\left(1-\exp\left(-k\cdot d_{99\%}\right)\right)$. Thus, it can be easily calculated that $d_{99\%}=-\ln\left(0.01\right)/k≅4.605/k$. The *d*_99%_ parameter gives an approximation of the electromagnetic wave penetration-depth calculated from digital simulations or VNA measurements. The summarized results of digital simulations for selected geometries of the tested probe for three values of $\varepsilon_{s}$ equal to 6, 20 and 80 were shown in Table 1.

The measured material volume was a cylindrical shell with the internal inner radius *R*_ext_ equal to the radius of the P-TDT supporting tube and *d*_99%_ thickness. The average value of *d*_99%_ calculated from digital simulations for three tested materials with the values of the static dielectric permittivity $\varepsilon_{s}$ equal to 6, 20 and 80 was *d*_99%_ave_ = 26.05 ± 1.40 (standard deviation of the sample mean) for the chosen geometry marked in bold in Table 1. It can also be seen that the smaller the distance between the strips, the smaller is the penetration depth. The value of *d*_99%_ for the distance between metallic strips *s* = 5 mm was significantly smaller than for longer distances, which were tested. It seems that the geometry *R*_ext_ = 20 mm, *s* = 15 mm was a reasonable choice for building the P-TDT prototype probe.

Since no change of the transmission time *t* was observed for various values of $EC_{b}$ for a fixed value of *d* (see Figure 7b), the parameters *A* and *k* in Formula (3) apply for all values of $EC_{b}$ in the analyzed range of variability from 0 to 10 dSm^−1^. Therefore, it was concluded that the penetration depth *d*_99%_ determined from the digital simulations did not depend on the bulk electrical conductivity $EC_{b}$ of the material surrounding the P-TDT probe.

An important issue in the presented calculations was the measurement range of the transmission time *t* described by parameter *A*, which refers to the infinite value of *d*. The value of *A* changes from 0.47 ns for $\varepsilon_{s}$ = 6 (dry soil) to 2.69 ns for $\varepsilon_{s}$ = 80 (water). So, the propagation time *t* measurement with a sufficient resolution in this time range, for example 1000 points in a linear time scale, gives about 2 ps spacing between two measurement points, which is an engineering challenge.

### 3.2. VNA Measurements on the P-TDT Prototype

**Figure 9:**
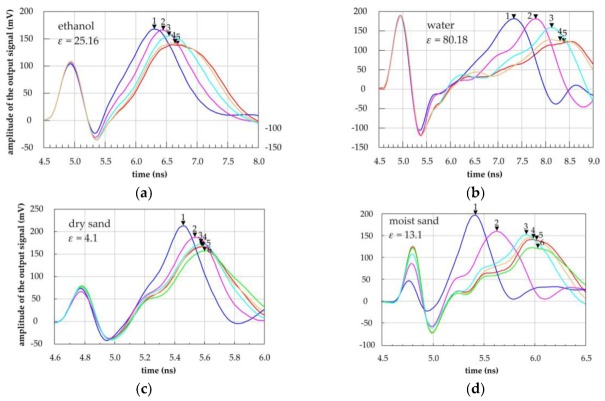
Time domain outputs from ADS electrical simulator with waveforms of a needle pulse transmitted along a section of the P-TDT probe prototype centrally inserted into plastic containers of internal diameters 50 mm, 56 mm, 66 mm, 83 mm and 103 mm filled with (**a**) ethanol; (**b**) distilled water and a P-TDT probe inserted into plastic containers of internal diameters 46 mm, 65 mm, 82 mm, 100 mm, 125 mm and 146 mm filled with (**c**) dry sand, *ε* = 4.1 and (**d**) moistened sand *ε* = 13.1.

The prototype P-TDT probe geometry was chosen on the basis of the digital simulation results (*R*_ext_ = 20 mm, *s* = 15 mm). Verification of the digital simulation for this construction was done with ethanol, distilled water and sandy soil as measurement media, and plastic containers and tubes with various diameters. The output waveforms in the time-domain that were generated by ADS electrical simulator on the base of the ***S*** scattering matrix measured by VNA, are presented in Figure 9. Similar waveforms could be produced directly by the VNA equipped with a time-domain conversion option. The filled triangles are markers showing the values of propagation time *t*, which are further processed to obtain the *d*_99%_ parameter as the P-TDT penetration depth indicator. The waveforms in Figure 7, where the scattering matrix ***S*** was determined with FEM 3D simulations, and the waveforms in Figure 9a–d, where ***S*** was measured by VNA connected to the prototype P-TDT probe, are very similar. The first pulse result from the signal crosstalk by the capacitive coupling between the beginning and the ending of the transmission line made of metallic strips. The other positive pulses on the waveform indicate the discontinuity of impedances between the transmission line on the surface of the P-TDT probe and the planar 50 ohm transmission line on the printed circuit board. Similarly to the digital simulations, the bigger diameter *d* of the container filled with ethanol, water or soil, the longer is the travel time *t* of the pulse along the P-TDT transmission line.

The graphical relation between travel time *t* and material thickness *d* for the prototype P-TDT probe is shown in Figure 8b,c. The parameters of the fitted exponential limit curve (3) are presented in Table 2.

The penetration depth (or the measured effective thickness) *d*_99%_ of the dielectric material measured by the P-TDT probe was bigger than the values received from digital simulations. Comparing the value of this parameter from the measured data on ethanol and water with the simulated data for the value of $\varepsilon_{s}$ = 20 and 80, we saw a 24% and 9% increase respectively. This effect can be explained by the possible errors in reading the travel time t from the waveforms in the time domain generated by the ADS electronic circuits simulation software for the measured data, especially for large diameter values of the containers with water (see Figure 9b). The penetration depth calculated from Formula (3) for sandy soil was 49.08 mm and 55.36 mm for dry and moist sand respectively. However, measurement data from Figure 8c showed that good enough measurement results for dry soil could be obtained even for a 20 mm thick sample and 30 mm for moist soil. Care should be taken to design and manufacture the interfaces between the successive elements along the fast pulse signal travel path to avoid signal reflections and distortions. They could significantly increase measurement error.

## 4. Conclusions

The combination of 3D FEM digital simulations for geometry of the P-TDT sensor has been presented. The simulations in frequency and time domain were performed. The ***S*** scattering matrix obtained from frequency domain described the interaction of a sensor with the surrounding material. Moreover, the ***S*** matrix was used in time domain simulations of the final electrical circuit of the sensor. The presented combination of 3D FEM digital simulations can significantly shorten the time for developing a working sensor prototype. This is especially useful for sensors working in the microwave frequency range, where mechanical imperfections introduce signal distortion.

The penetration depth evaluated by the digital simulations and verified by VNA measurements on the prototype P-TDT probe appeared to be sufficient for the practical applications for soil moisture measurements in the frequency range comparable to the commonly used TDR technique. The material thickness measured by the analyzed soil moisture profile probe, as defined by the *d*_99%_ indicator, was in the range of 20–30 mm. It did not depend on soil electrical conductivity in the range up to 5 dSm^−1^, which corresponds to the commonly accepted soil bulk electrical conductivity upper limit for plant growth.

The transmitted signal amplitude corresponded to the soil bulk electrical conductivity, which enabled simultaneous measurements of soil moisture and salinity from the same soil volume. The addition of a temperature sensor in close proximity to the metallic strips of the P-TDT section would also be necessary to make temperature corrections of the measured electric pulse time propagation. This propagation time enables soil moisture to be calculated, but it depends on dielectric permittivity, which in turn depends on temperature. It might be problematic to measure very short time distances in the time scale of the fractions of a nanosecond with an acceptable resolution and accuracy, which could affect the VWC measurement accuracy of dry soil. Future work on the electronic hardware and software will address these issues with short time distances.

In this paper, the initial part of the research on the P-TDT probes was presented. It is expected that the final product will be an economic and durable field measurement device for monitoring the dynamics of soil moisture, electrical conductivity and temperature at various soil layers. The performance of the final construction of the P-TDT probe, including the applied electronic solutions, is to be the subject of a future paper.

## Figures and Tables

**Table 1:** Selected values of fitting parameters *A* and *k* from formula (3) together with calculated values of the penetration depth indicator *d*_99%_ for digitally simulated data of the P-TDT probe.

*R*_ext_ (mm)	Param.	$\varepsilon_{s}\;=\;6$				$\varepsilon_{s}\;=\;20$				$\varepsilon_{s}\;=\;80$			
		*s* (mm)				*s* (mm)				*s* (mm)			
		5	10	15	20	5	10	15	20	5	10	15	20
17	*A* (ns)	0.34	0.38	0.40	-	0.87	0.95	1.01	-	2.03	2.21	2.35	-
	*k* (mm^−1^)	0.35	0.26	0.22	-	0.38	0.27	0.23	-	0.39	0.27	0.24	-
	*d*_99%_ (mm)	13.12	17.80	20.59	-	12.26	16.84	19.79	-	11.89	16.98	19.44	-
20	*A* (ns)	0.37	0.44	0.47	0.47	0.93	1.13	1.19	1.33	2.20	2.61	2.69	2.65
	*k* (mm^−1^)	0.31	0.20	0.17	0.17	0.31	0.22	0.18	0.23	0.34	0.23	0.18	0.19
	*d*_99%_ (mm)	14.82	22.66	**27.67**	26.32	14.69	21.35	**25.13**	19.88	13.70	20.04	**25.36**	24.83
25	*A* (ns)	-	-	0.53	0.53	-	-	1.36	1.39	-	-	3.15	3.21
	*k* (mm^−1^)	-	-	0.20	0.20	-	-	0.21	0.17	-	-	0.22	0.18
	*d*_99%_ (mm)	-	-	22.78	22.69	-	-	21.48	27.63	-	-	20.71	26.11

**Table 2:** Parameters of the fitted exponential limit curve (4) and calculated values of the penetration depth indicator *d*_99%_ values for ethanol, distilled water and sandy soil for P-TDT probe prototype.

Fitted Parameters	Material Values of *ε_s_* Used for VNA Measurements			
	25.16 (Ethanol)	80.18 (Distilled Water)	4.1 (Dry Sand)	13.1 (Moist Sand)
*A* (ns)	0.67	2.10	0.27	0.86
*k* (mm^−1^)	0.15	0.17	0.14	0.07
*d*_99%_ (mm)	31.09	27.67	32.05	67.05
